# Subcutaneous enucleation of the radial neck in a Monteggia-like fracture: an exceptional variant

**DOI:** 10.1016/j.ijscr.2025.111866

**Published:** 2025-08-24

**Authors:** Herijaona Manasse Rakotoherisoa, Thomas Daoulas, Anselme Als Christophane Anesy, Pierre Maincourt, Olivier Bozon, Henri Jean Claude Razafimahandry

**Affiliations:** a CHU Joseph Ravoahangy Andrianavalona, Service de Chirurgie Orthopédique et Traumatologique, 101 Tananarive, Madagascar; b CHU Cavale Blanche, Service de Chirurgie Orthopédique et Traumatologique de Brest, Boulevard Tanguy Prigent, 29200 Brest, France; c CHU Cavale Blanche, Service de Chirurgie plastique reconstructrice et esthétique, Boulevard Tanguy Prigent, 29200 Brest, France; d Orthopedic Surgery Department, Upper Limb, Hand, and Peripheral Nerve Surgery Unit, Lapeyronie University Hospital, 191 Avenue of Doyen Gaston Giraud, 34090 Montpellier, France

**Keywords:** Bado classification, Monteggia fracture, Radial head

## Abstract

**Introduction and importance:**

Monteggia lesions combine a fracture of the ulna with dislocation of the radial head. Monteggia-like variants add a radial head fracture. We report a unique Monteggia-like injury with a bifocal radial fracture and subcutaneous enucleation of the radial neck, which does not fit existing classifications.

**Case presentation:**

A 29-year-old male sustained a high-energy axial trauma to the flexed, supinated left elbow. Examination showed isolated swelling without neurovascular injury. X-rays revealed a transverse ulnar fracture with a multifragmentary radial head fracture and a bifocal metaphyso-diaphyseal radial fracture. The intermediate radial fragment was displaced posterolaterally into subcutaneous tissue. Surgical treatment included ORIF of the ulna and excision of the subcutaneous radial fragment; the radial head was left in place under the capitellum. Postoperative care involved immobilization for three weeks with early mobilization. At two years, the patient had minimal pain but marked limitation in rotation (70° pronation, 30° supination); flexion-extension was 0–130°, and the DASH score was 32/100.

**Clinical discussion:**

No similar fracture pattern has been reported. The injury likely resulted from an unusual combination of significant elbow flexion and full supination under axial load, preserving the annular ligament and radial head position. Reconstruction of the radial neck was not possible due to comminution, risk of nonunion, and lack of hardware. Importantly, the treatment context involved significant technical limitations influencing the surgical strategy. Functional limitation highlights the importance of restoring radial neck anatomy to maintain supination and pronation.

**Conclusion:**

This rare variant underscores the need for awareness of atypical Monteggia-like patterns and the importance of anatomical reconstruction for optimal functional recovery.

**Consent:**

Written informed consent was obtained from the patient for publication and any accompanying images. A copy of the written consent is available for review by the Editor-in-Chief of this journal on request.

## Highlights

• Rare Monteggia-like lesion with subcutaneous enucleation of the radial neck.
• High-energy axial trauma with elbow in 120° flexion and maximal supination.
• Radial head preserved beneath intact annular ligament; neck avulsed posterolaterally.
• Surgical strategy constrained by hardware unavailability; radial neck fragment excised.
• Long-term outcome: preserved flexion-extension but major pronation-supination deficit.

## 1 Introduction

Monteggia lesions combine a fracture of the ulna with dislocation of the radial head. Monteggia-like fractures also involve a fracture of the radial head [1]. They are traditionally classified according to Bado, based on the direction of radial head displacement and the location of the ulnar fracture [2]. The Jupiter modification of Bado type II allows differentiation into four subtypes based on the location of the ulnar fracture [3].

We report an unusual Monteggia-like fracture that does not fit into any of these classifications.

## 2 Observation

The patient was a 29-year-old male construction engineer who sustained a high-energy direct trauma to the left upper limb. The mechanism involved an axial load directed from distal to proximal, with the elbow flexed at approximately 120° and the forearm in maximal supination (90°). Clinical examination revealed a swelling located 2 cm above the posterior border of the left lateral epicondyle, without any skin breach or neurovascular complications, particularly involving the ulnar and radial nerves.

**Fig. 1:**
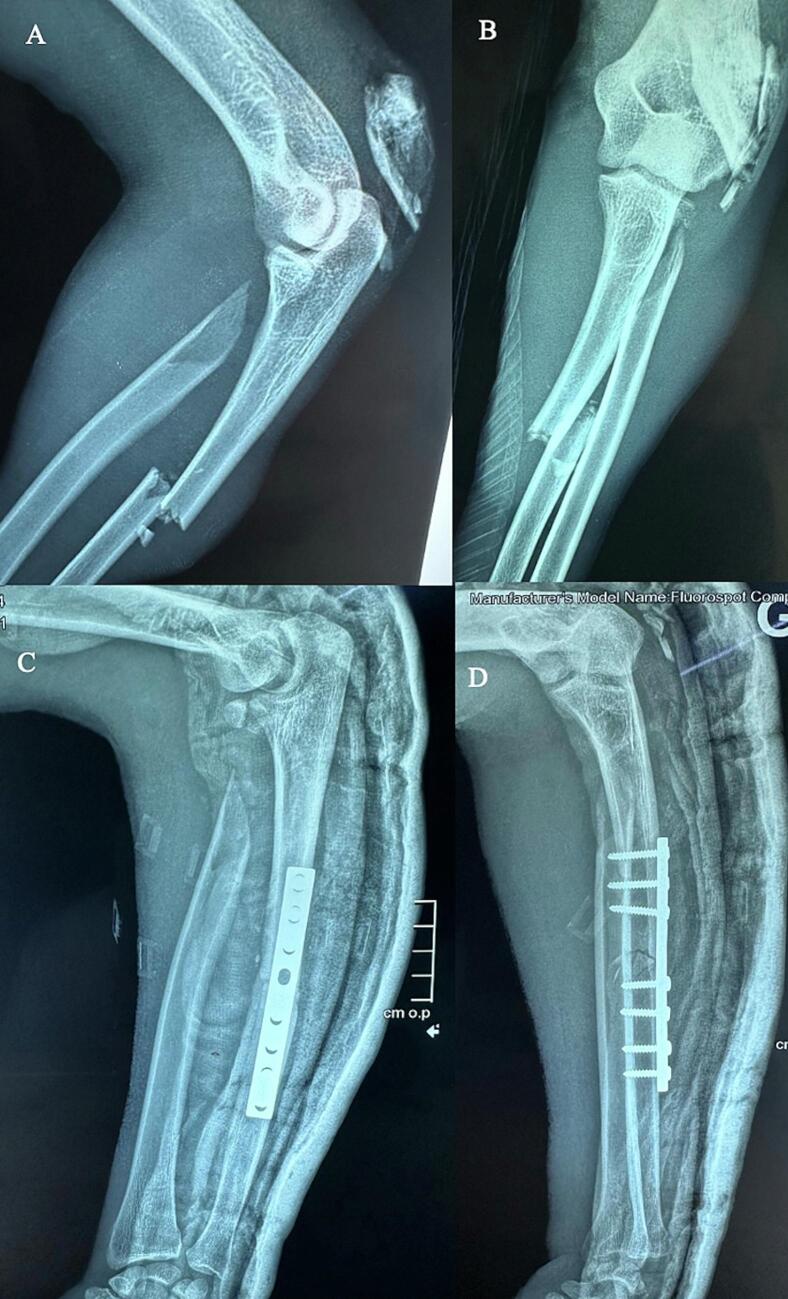
(A–B) Preoperative images showing a variant of a Monteggia fracture with enucleation of the radial neck to the posterolateral aspect of the elbow. (C–D) Postoperative images demonstrating open reduction and internal fixation of the ulnar fracture with a DCP plate and excision of the radial neck fragment.

Initial radiographic assessment demonstrated a transverse fracture at the junction of the proximal and middle third of the ulnar diaphysis, associated with a multifragmentary fracture of the radial head and a bifocal metaphyso-diaphyseal fracture of the proximal radius. The intermediate fragment was displaced posterolaterally into the subcutaneous tissue (Fig. 1).

Although the radial head was not dislocated per se, we considered this injury to be a Monteggia fracture variant based on the underlying biomechanical mechanism. Specifically, the radial neck absorbed the dislocating force component through a compression mechanism superimposed on the classical posterior Monteggia lesion mechanism. Therefore, despite the absence of a frank dislocation of the radial head, the injury functionally corresponded to a Monteggia fracture where the radial neck played a key role in dissipating the dislocation forces.

Surgical management consisted of open reduction and internal fixation (ORIF) of the ulna using a 3.5 mm DCP plate. The radial head was preserved beneath the capitellum, supported by the intact annular ligament. The subcutaneous fragment of the radial neck was excised through a direct posterolateral approach over the swelling. No exploration of potential ligamentous injuries was performed.

Postoperative care included immobilization with a removable above-elbow splint for three weeks, allowing immediate initiation of active rehabilitation.

**Fig. 2:**
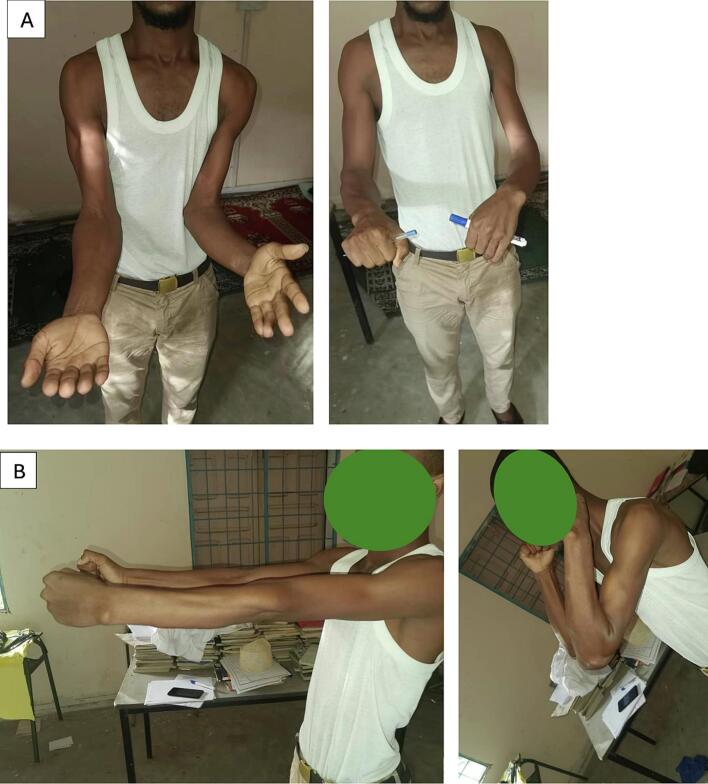
Clinical examination at two-year follow-up: (A) Pronosupination range of motion showing limited supination to 30°. (B) Satisfactory flexion–extension.

At two-years follow-up, the patient reported near-complete resolution of pain (VAS 1/10) but had limited forearm rotation with 70° of pronation and 30° of supination. Elbow flexion-extension range of motion was 0–130° (Fig. 2). The DASH score was 32/100.

## 3 Discussion

### 3.1 Concerning the fracture and the mechanism

**Fig. 3:**
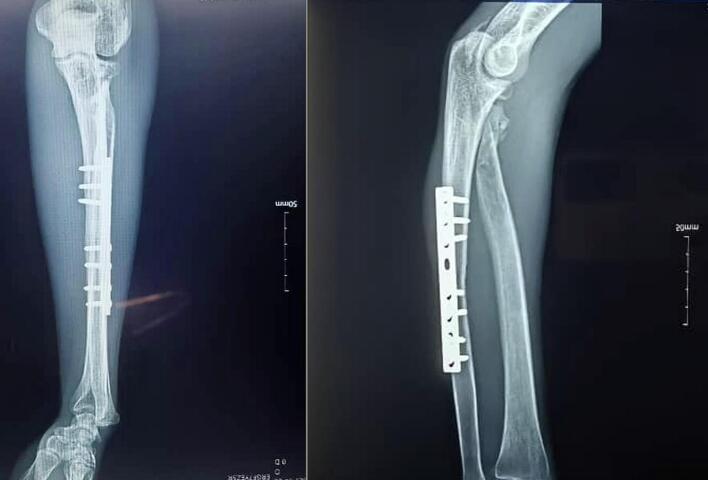
Postoperative radiographs at final follow-up: fracture healing achieved. Major heterotopic ossifications. The radial head is healed and encapsulated within the intact annular ligament.

No description of this type of fracture has been found in the literature. The reported injury could resemble a Bado type II Monteggia-like lesion, defined as a posterolateral dislocation of the radial head associated with a fracture of the ulnar diaphysis. However, in this case, the radial head remained positioned beneath the capitellum, likely held in place by an intact annular ligament. The dislocation component thus involved the radial neck, which avulsed posterolaterally along with the bicipital tuberosity and became lodged in the subcutaneous tissue (Fig. 3).

This exceptional variant may be explained by the unusual biomechanical context, namely 90° of forearm supination and significant elbow flexion at the time of the axial load applied from distal to proximal. Typically, in such trauma mechanisms, the forearm is only slightly flexed during axial compression. A high-energy axial load with the elbow flexed beyond 90° is rare.

In addition to bony injuries, Monteggia and Monteggia-like lesions also involve soft tissue damage, particularly affecting the annular ligament [4]. In this case, the annular ligament remained intact, maintaining the radial head in its anatomical position despite the fracture. The force vector appears to have propagated primarily through the bicipital tuberosity.

The complexity of the lesion described here makes it difficult to classify within the traditional Bado or Jupiter systems. Several authors have reported Monteggia-like variants that do not conform to these classifications [[5], [6], [7]]. However, to our knowledge, no case in the literature has reported such an enucleation of the radial neck into the subcutaneous tissue in the context of a Monteggia lesion.

### 3.2 Concerning the fracture and the mechanism

The primary objective in the management of Monteggia-type lesions is the anatomical reduction of the ulna [8]. In cases of associated radial fractures, most authors recommend preserving the native radial head whenever possible [1,8]. In cases of highly complex injuries, radial head arthroplasty is generally indicated [9,10]. However, some authors have reported favorable outcomes following radial head resection in the context of Monteggia-like injuries [1]. The case we report was not eligible for radial head arthroplasty due to the metaphyseal-diaphyseal extension of the radial fracture. Open reduction and internal fixation (ORIF) of the radial fracture was considered; however, the significant comminution at the fracture site posed a high risk of nonunion or avascular necrosis of the proximal epiphysis. This risk, combined with the unavailability of sufficiently long plates for stable fixation and the lack of necessary implants for radial head arthroplasty in the country where the patient was treated, led us to select an alternative therapeutic approach. Moreover, all surgical costs, including consumables, were directly borne by the patient, who could not afford such expenses. Consequently, excision of the radial neck fragment with preservation of the radial head in situ was chosen as a compromise dictated by both material and financial constraints. While arthroplasty or delayed reconstruction might offer better biomechanical and functional outcomes in well-resourced settings, these options were not accessible here, underscoring the challenges of managing complex Monteggia-like injuries in low-resource environments.

At two-years follow-up, the functional outcomes were mixed, primarily due to the significant limitation in the pronation-supination range of motion. The intraoperative decision to leave the radial head in place within the annular ligament is debatable in retrospect. In the absence of the radial neck, the head no longer plays its stabilizing role against valgus stress and may have contributed to stiffness through circumferential calcification of the region. The patient's DASH score was 32/100, reflecting substantial difficulties in performing recreational activities requiring strength with the affected limb, challenges in carrying heavy objects, and limitations impacting work-related tasks.

The severe limitation in supination can be attributed to the lack of anatomical restoration of the radial neck. The avulsed radial neck fragment included the entire bicipital tuberosity and the majority of the distal insertion of the supinator muscle. As both primary supination motors were rendered nearly non-functional, a poor functional result in supination was anticipated given the anatomical disruption. The restriction in pronation, on the other hand, is likely due to major adhesions and fibrosis in the affected area.

Anatomical reconstruction of the radial neck and head might have yielded better functional results. The decision not to pursue a conservative treatment was also influenced by the lack of appropriate surgical hardware in the country where the procedure was performed, which precluded internal fixation for this fracture type.

Our functional outcomes support existing literature emphasizing the need to preserve the anatomical relationships of the forearm ring to restore the most physiological possible kinematics during pronation-supination movements.

The work has been reported in line with the SCARE criteria [11].

## 4 Conclusion

We report a novel type of Monteggia-like lesion, combining an avulsion fracture of the radial neck displaced subcutaneously to the posterolateral aspect of the elbow. Functional outcomes following ulnar osteosynthesis and excision of the avulsed neck fragment were mixed.

## CRediT authorship contribution statement

• **T.D.:** Methodology – Writing
• **H.M.R.:** Original version – Conceptualization
• **A.A.C.A.:** Resources
• **P.M:** Validation
• **O.B.:** Writing
• **H.J.C.R.:** Project administration, Writing – Review and editing

## Consent

Written informed consent was obtained from the patient for publication and any accompanying images. A copy of the written consent is available for review by the Editor-in-Chief of this journal on request.

## Ethical approval

Ethical approval for this study (Ethical Committee N°345 MSANP/SGIDGFS/HJRA/DE) was provided by the Ethical Committee of CHU JRA, Antananarivo, Madagascar, on 24 January 2025.

## Guarantor

Thomas Daoulas.

## Declaration of Generative AI and AI-assisted technologies in the writing process

All authors certify that they have not used AI in the writing, data collection and design of this article.

## Sources of funding

None.

## Declaration of competing interest

The authors declare no conflict of interest.
